# Association between the Inflammatory Potential of the Diet and Biological Aging: A Cross-Sectional Analysis of 4510 Adults from the Moli-Sani Study Cohort

**DOI:** 10.3390/nu15061503

**Published:** 2023-03-21

**Authors:** Claudia F. Martínez, Simona Esposito, Augusto Di Castelnuovo, Simona Costanzo, Emilia Ruggiero, Amalia De Curtis, Mariarosaria Persichillo, James R. Hébert, Chiara Cerletti, Maria Benedetta Donati, Giovanni de Gaetano, Licia Iacoviello, Alessandro Gialluisi, Marialaura Bonaccio

**Affiliations:** 1 Department of Epidemiology and Prevention, IRCCS Neuromed, Via dell’Elettronica, 86077 Pozzilli, Italy; 2 Population Health Research Center, National Institute of Public Health, Cuernavaca 62100, Mexico; 3 Mediterranea Cardiocentro, 80122 Napoli, Italy; 4 Cancer Prevention and Control Program and Department of Epidemiology and Biostatistics, Arnold School of Public Health, University of South Carolina, Columbia, SC 29208, USA; 5 Department of Nutrition, Connecting Health Innovations LLC, Columbia, SC 29201, USA; 6 Department of Medicine and Surgery, Research Center in Epidemiology and Preventive Medicine (EPIMED), University of Insubria, 21100 Varese-Como, Italy

**Keywords:** aging, biological age, inflammation, inflammatory diet

## Abstract

Chronological age (CA) may not accurately reflect the health status of an individual. Rather, biological age (BA) or hypothetical underlying “functional” age has been proposed as a relevant indicator of healthy aging. Observational studies have found that decelerated biological aging or Δage (BA-CA) is associated with a lower risk of disease and mortality. In general, CA is associated with low-grade inflammation, a condition linked to the risk of the incidence of disease and overall cause-specific mortality, and is modulated by diet. To address the hypothesis that diet-related inflammation is associated with Δage, a cross-sectional analysis of data from a sub-cohort from the Moli-sani Study (2005–2010, Italy) was performed. The inflammatory potential of the diet was measured using the Energy-adjusted Dietary Inflammatory Index (E-DII^TM^) and a novel literature-based dietary inflammation score (DIS). A deep neural network approach based on circulating biomarkers was used to compute BA, and the resulting Δage was fit as the dependent variable. In 4510 participants (men 52.0%), the mean of CA (SD) was 55.6 y (±11.6), BA 54.8 y (±8.6), and Δage −0.77 (±7.7). In a multivariable-adjusted analysis, an increase in E-DII^TM^ and DIS scores led to an increase in Δage (β = 0.22; 95%CI 0.05, 0.38; β = 0.27; 95%CI 0.10, 0.44, respectively). We found interaction for DIS by sex and for E-DII^TM^ by BMI. In conclusion, a pro-inflammatory diet is associated with accelerated biological aging, which likely leads to an increased long-term risk of inflammation-related diseases and mortality.

## 1. Introduction

Aging is a complex process that results from a wide variety of molecular and cellular damage over time that therefore varies across individuals [1]. Globally, the proportion of people aged over 60 years is increasing, thus placing burdens on health systems across the world [2]. In unhealthy aging, “inflammaging”, defined as low-grade chronic inflammation in the absence of known infections or other established causes, occurs [3]. Inflammaging constitutes a marker of accelerated aging and increased morbidity [4,5,6] and disability [7]. Several mechanisms are involved, including the accumulation of cellular damage [8], changes in the gut and oral microbiota [9], and cellular senescence [10], which causes an increase in inflammatory cytokines, particularly in visceral fat [11]. Chronological age (CA) is limited in capturing the heterogeneity of aging events and their impact on health. The concept of biological age (BA)—namely, the actual underlying biologically relevant age of an organism—has been proposed to provide a better understanding of the heterogeneity of the aging process across individuals. BA can be estimated through multiple algorithms and biomarkers [12,13,14]. The resulting discrepancy between BA and CA is usually indicated by Δage, which may suggest either accelerated (Δage > 0) or decelerated biological aging (Δage < 0) [13]. Negative values of Δage (i.e., where BA is less than CA) are associated with the deceleration of aging and a lower risk of morbidity, hospitalization, and mortality [15,16]. One of the most innovative ways to estimate biological aging is by applying deep neural networks to circulating biomarkers [16,17,18,19]. Indeed, although this represents only a generic marker of biological aging and other markers or scales such as frailty and cognitive performance may better tag organ-specific aging [20] or the intrinsic aging capacity [21,22], blood-based estimates of BA can provide information on several aging domains within the human body because it can be based on a range of different circulating biomarkers. Indeed, previous studies identified prominent roles of glucose homeostasis, liver and kidney functionality, and inflammation, among other biomarkers [16,17,18,19]. Moreover, the wide availability of routine blood tests resulting from common clinical practice makes this a cost-effective estimator of biological aging, which could be used as a public health and healthy aging screening tool in the general population [16].

Despite evidence suggesting a prominent role of healthy dietary patterns in modulating healthy aging, the association between dietary exposures and biological aging parameters remain understudied. However, previous observations suggest a central role of diet in the regulation of subclinical inflammation, a precursor of chronic diseases [23,24], which is also inherently linked to inflammaging [3]. Plant-based, whole-food dietary patterns characterized by food rich in compounds with anti-inflammatory activity, e.g., the Mediterranean diet, appear to promote healthy aging [25]. By contrast, a pro-inflammatory diet leads to low-grade inflammation and, consequently, an increased risk of chronic conditions, such as cancer, metabolic disorders, and depressive symptoms [23,26,27]. The dietary inflammatory index (DII^®^) [27]; and the energy-adjusted version (E-DII^TM^) [28] are literature-based tools widely used to assess the inflammatory potential of the diet associated with health outcomes including cancer, cardiovascular diseases, adverse mental health, cardiometabolic risk, and frailty [29,30,31,32,33,34]. The DII, which is based on existing literature, includes up to 45 food parameters, including 35 nutrients and 10 whole foods. Many data sets do not include whole foods because programs often compute and output only nutrients; so, most will have fewer than 45 parameters. Because the E-DII includes energy in the denominator, it will have one fewer parameter than the DII. Because, in most datasets, the DII is based on nutrients, it might be useful to consider additional whole foods that contain multiple interacting substances and nutrients [35]. To address this concern, Byrd et al. developed and validated a novel FFQ-based dietary inflammation score (DIS) that includes whole foods, beverages, and micronutrient supplements. In a validation study within three populations, the use of the DIS suggests stronger associations with plasma inflammation biomarkers than DII [36]. Moreover, a pro-inflammatory DIS value has been associated with all-cause mortality [37] and with an increased risk of colorectal cancer [38]. It should be noted that with over 900 publications, the DII/E-DII literature is much more robust [36].

We performed a cross-sectional analysis in a sub-cohort from the Moli-sani Study (2005–2010, Italy) to examine the potential association of pro-inflammatory diets with biological aging. We hypothesized that a proinflammatory diet is directly associated with accelerated biological aging, estimated using a blood-based deep learning algorithm.

## 2. Methods

### 2.1. Study Population

We analyzed data from the Moli-sani Study, a large population-based cohort designed to investigate genetic and environmental risk factors associated with cardiovascular and cerebrovascular diseases and cancer. At the baseline survey performed between 2005 and 2010, 24,325 subjects (aged ≥ 35 years) were recruited from city-hall registries of the Molise region. Exclusion criteria were pregnancy at the time of recruitment, mental impairments, current poly-traumas or coma, or refusal to sign the informed consent form. The Moli-sani Study complies with the Declaration of Helsinki and was granted the approval of the Ethics Committee of the Catholic University in Rome, Italy. Additional details of the study design are available elsewhere [39]. For the present analyses, we excluded individuals with missing data on diet (n = 20) or with implausible energy intake (<800 or >4000 kcal/d in men; <500 or >3500 kcal/d in women) (n = 126), or individuals with medical (n = 43) or dietary questionnaires judged as unreliable (n = 179).

### 2.2. Computation of Biological Age

To compute biological age, we used a supervised machine learning algorithm called a deep neural network (DNN). From the initial 24,325 participants, Δage was calculated in a test set of 4772 subjects as described below [16]. We deployed a DNN for the prediction of BA using 36 circulating biomarkers, using recruiting center and sex as input features, and the CA of each participant as a label. Biomarkers included were (a) glucose metabolism: glucose, C-peptide, and insulin; (b) lipids: triglycerides, high and low-density lipoprotein-cholesterol, lipoprotein a and apolipoprotein A1 and B; (c) liver enzymes: aspartate transaminase and alanine aminotransferase; (d) renal function: uric acid, albumin, creatinine, cystatin-C; (e) vascular and cardiac: NT-proB-type Natriuretic Peptide and high-sensitivity cardiac troponin I; (f) hormones: testosterone and vitamin D; (g) hemostasis: D-Dimer; (h) inflammation: high-sensitivity C-reactive protein; (i) haemachrome: red blood cell count and distribution width, hematocrit, hemoglobin levels, mean corpuscular volume, mean corpuscular hemoglobin concentration, total white blood cells, lymphocytes, monocytes, granulocytes, neutrophils, basophils, and eosinophils; platelet count, mean platelet volume, and platelet distribution width. The DNN was built in R v3.9 through the Keras package v2.4.0 (https://www.r-project.org/; https://cran.r-project.org/web/packages/keras/index.html; accessed on 15 September 2022). We split the available dataset passing quality control (n = 23,858) into a random training and test set (80:20 ratio), then trained the algorithm over 1000 epochs in the training set and evaluated the accuracy in the test set. For each participant, BA and the resulting discrepancy with CA were computed (∆age = BA–CA) within the training set (n = 4772), which was used within the study population (i.e., the test set. A permutation feature importance analysis revealed that the most influential features on BA (hence ∆age) estimates—namely those showing a loss-drop after permutations of at least 5% compared to the original non-permuted model—were cystatin-C, NT-proBNP, sex, creatinine, glucose, ALT, AST, triglycerides and D-Dimer [16]. Other details on quality control, DNN architecture, and performance are reported elsewhere [16]. The final analysis was carried out in the remaining test sample of 4510 subjects after applying the exclusion criteria mentioned above.

### 2.3. Dietary Assessment

Food intake during the year before enrolment was assessed through an interviewer-administered EPIC 188-item food frequency questionnaire (FFQ) [40], which was validated and adapted to the Italian population. The food items were classified into 45 predefined food groups based on similar nutrient characteristics or culinary usage. Frequencies and quantities of each food were linked to Italian Food Tables using specialized software [41,42] to estimate energy, macro-, and micro-nutrient intake.

### 2.4. Computation of DII and E-DII Scores

We calculated the DII and E-DII scores for all subjects using FFQ-derived dietary information, as mentioned above and described in detail elsewhere [27,28]. The dietary data for each study participant were first linked to the regionally representative global database that provided a robust estimate of a mean and standard deviation for each of the food parameters (i.e., foods, nutrients, and other compounds such as flavonoids). A z-score was derived by subtracting the “standard global mean” from the amount reported and dividing this value by the standard deviation (SD). The z-score was converted to a centered proportion and then multiplied by the respective food parameter inflammatory effect score (derived from a literature review and scoring of 1943 “qualified” articles) to obtain the subject’s food parameter-specific DII score. To compute the overall DII score for every subject in the study, all the food parameter-specific DII scores were summed. We repeated this procedure for the E-DII using calorie-adjusted values for intake and using a calorie-calorie-adjusted global comparative database to compute Z scores and, ultimately, the overall E-DII score. For the current analysis, data were available for a total of 34 food parameters (carbohydrate, protein, total fat, alcohol, fiber, cholesterol, saturated fat, monounsaturated fat, polyunsaturated fat, omega-3, omega-6 fatty acid, niacin, thiamin, riboflavin, vitamin B12, vitamin B6, iron, magnesium, zinc, vitamin A, vitamin C, vitamin D, vitamin E, folic acid, β-carotene, anthocyanidins, flavan-3-ols, flavones, flavanols, flavonones, isoflavones, garlic, onion, tea). 

The DIS was calculated using the method described by Byrd et al. [36], consisting of 19 food groups (18 whole foods and beverages and 1 composite micronutrient supplement group) that were selected *a priori* based on biological plausibility and previous literature (Supplemental Table S1). The DIS components (dietary and supplemental intakes) were acquired from FFQ used in our cohort [40]. An individual’s DIS score was then calculated as the sum of their weighted components. For comparison purposes, both scores were standardized.

### 2.5. Ascertainment of Covariates

Information about sociodemographic factors, lifestyles, and clinical variables was obtained at baseline via interviewer-administered questionnaires. Personal history of cancer and cardiovascular disease (angina, myocardial infarction, revascularization procedures, peripheral artery diseases, and cerebrovascular events) and drug treatment were self-reported and confirmed by medical records. Participants were considered to have hypertension, hyperlipidemia, or diabetes at baseline if they reported having been treated with disease-specific drugs. Leisure-time physical activity (PA) was expressed as daily energy expenditure in metabolic equivalent task hours (MET-h/d) for sport, walking, and gardening. Height and weight were measured, and body mass index (BMI) was calculated as weight (kg)/height (m)^2^ and grouped into three categories normal (≤25 kg/m^2^), overweight (>25 < 30 kg/m^2^), or obese (≥30 kg/m^2^). Subjects were classified as never, current, or former smokers (reported not having smoked at all over the previous 12 months or more). Education was based on the highest qualification attained and was categorized as up to lower secondary (approximately ≤8 years of study), upper secondary school (9–13 years of study), and post-secondary education (>13 years of study). Housing tenure was classified as rented, ownership of one dwelling, and ownership of more than one dwelling. Urbanization was classified as living in either an urban or rural area based on the urbanization level (defined by the European Institute of Statics, EUROSTAT) and obtained by the tool “Atlante Statistico dei Comuni” provided by the Italian National Institute of Statistics [43].

### 2.6. Statistical Analysis

Characteristics of the study population are presented as number and percentage or mean and standard deviation (±SD) for continuous variables. 

Multivariable-adjusted linear regression models were fit to estimate β-coefficients and corresponding 95% confidence interval (95% CI) for the relation between the E-DII and the DIS scores (independent variables, scores were standardized for comparison purposes) with Δage (PROC REG in SAS). Missing values for covariates, i.e., history of CVD (n = 68), cancer (n = 19), diabetes (n = 62), hyperlipidemia (n = 45), hypertension (n = 42), menopausal status (n=6), education (n = 1), housing (n = 3), smoking habits (n = 5), hormone replacement therapy (n = 102), leisure-time PA (n = 42) and BMI (n = 4) were handled using a multiple imputation technique (SAS PROC MI and PROC MIANALYZE). To maximize data availability for all variables and to avoid bias introduced by data not missing-at-random, multiple imputation was performed (n = 10 imputed datasets). Potential confounders were defined a priori based on the literature on associations with both diet and biological age [44,45]. Two models were fit: one with just age, sex, and energy intake adjusted l (not for analyses with E-DII); and a multivariable model additionally adjusted for education, housing, urban, leisure-time physical activity, smoking habit, BMI, CVD, cancer, diabetes, hypertension, hyperlipidemia, menopausal status, and hormone replacement therapy. In sensitivity analysis, we removed one comorbidity at a time from the principal model. Several subgroup analyses were conducted to test the robustness of the findings according to potential effect modification factors: age, sex, BMI (normal weight, overweight and obese), smoking status, and comorbidity [44]. 

We tested interaction using multiplicative terms. Statistical tests were two-sided, and P values of less than 0.05 were considered to indicate statistical significance. Data analyses were generated using SAS/STAT software, version 9.4 (SAS Institute Inc., Cary, NC, USA).

## 3. Results

The analytical sample consisted of 4510 participants (men 52.0%) with a proportion of 52.8% of participants in the lower education level and 63.1% with no comorbidities. The average ± SD of the biological age of participants was 54.8 ± 8.6 y, CA 55.6 ± 11.6 y, and Δage −0.77 ± 7.7. At baseline, the median score (interquartile range; IQR) for E-DII ^TM^ was 1.5 (0.2–1.6), and for DIS, −0.12 (−0.6–0.6); higher E-DII or DIS indicate a more proinflammatory diet. Participants in the higher quartile of E-DII and DIS were more likely to have no comorbidities and normal weight than those in the lowest quartile. Daily energy intake and macronutrients were similar across quartiles of the inflammatory potential of diet scores. On average, participants with more pro-inflammatory diets, according to DIS or E-DII, had a lower intake of fiber, fruits, and vegetables per day (Table 1). 

In the multivariable-adjusted analyses, an increase in the E-DII score was associated with acceleration in Δage (β = 0.22; 95%CI 0.05, 0.38). For DIS, we observed the same direction, although slightly greater magnitude (β = 0.27; 95%CI 0.10, 0.44) (Table 2). 

Subgroup analyses confirmed the association of pro-inflammatory diet and acceleration of biological aging only for DIS by sex: men (β = 0.08; 95%CI—0.17, 0.33); women (β = 0.43; 95%CI 0.21, 0.65); *p*-value for interaction = 0.03. We found an interaction between E-DII and BMI, participants with normal weight had an increase in acceleration of biological aging (β = 0.27; 95%CI—0.05, 0.60 *p*-value for interaction = 0.001). Increased accelerated aging was also observed among smokers when we analyzed DIS (β = 0.58; 95%CI 0.24, 0.93), although the *p*-value for interaction was not significant (0.16; Table 3). When we excluded comorbidities, the associations remained similar (Supplemental Table S2). In sensitive analyses with two cut-offs of age >65 and >70 years, the association was apparently weaker in older compared to younger groups (Supplemental Table S3).

## 4. Discussion

In a large Italian cohort of adults, a positive association was observed between pro-inflammatory diets and biological aging, as measured by a deep learning-based assessment based on many circulating biomarkers. The findings suggest that a large proportion of foods with high pro-inflammatory potential may promote an acceleration of aging, which is an independent risk factor for numerous chronic diseases and mortality [30,31,33,34,46]. Inflammation underlies many different biological aging clocks, even those not strictly based on inflammatory markers, as supported by recent bioinformatic evidence showing an association of inflammation-related gene products in aging-related molecular networks [47]. In line with this evidence, our deep learning aging clock was only partly based on inflammatory or inflammation-related markers [16]. Therefore, the findings reported here suggest that a pro-inflammatory diet may influence aging-related biological pathways (or molecular networks) not strictly related to the inflammatory response. Moreover, sensitivity analysis revealed that older subjects (above the age of 65 or 70 years) show a notably reduced association between inflammatory potential of diet and biological aging, suggesting that adopting healthful diets at an early age may be critical to reducing the future burden of aging. Further longitudinal studies are warranted to ascertain this hypothesis. 

Our results are in accordance with prior observations where a high adherence to well-known anti-inflammatory dietary patterns (e.g., Mediterranean Diet and DASH) and dietary polyphenols consumption were associated with delayed biological aging [48,49]. In cross-sectional studies, a pro-inflammatory diet, as reflected by a higher DII/E-DII score, was associated with increased levels of inflammatory markers [50] and metabolic syndrome [51]. Moreover, in a middle-aged Korean cohort, a vegetable-based dietary pattern rich in anti-inflammatory foods was inversely associated with a higher level of C-reactive protein, a biomarker of persistent low-grade inflammation [52]. In our study, the E-DII, based mainly on nutrients (as that is where the evidence exists in the biomedical literature), and the DIS, which is based exclusively on foods, were both associated with biological aging [36].

Biological aging is defined as an increased state of cellular vulnerability characterized by senescence, mitochondrial dysfunction, genomic and epigenomic instability, and telomere shortening. Telomere shortening is an important cause of stem cell decline in aging in multiple tissues [1]. In a 5-year longitudinal study on the Mediterranean diet (PREDIMED), a pro-inflammatory diet assessed through the DII was associated with telomere length [53]. In a 5-year longitudinal study (PREDIMED), a more pro-inflammatory diet, assessed using the DII, was associated with telomere shortening [53,54] In a cross-sectional study, higher adherence to a healthy diet with the DASH approach may be involved with slower epigenetic age acceleration [55]. In addition, in a pilot randomized clinical trial, plant-centered diet and lifestyle interventions, including relaxation techniques and exercise, may have a role in decreased epigenetic age [56].

The mechanisms underlying diet-related inflammation and its link with biological aging are still unclear. An unhealthy microbiota and its metabolites possibly are involved in the acceleration of age-related decline and the occurrence of an extensive number of diseases [57]. Moreover, reduced gut microbiota in older adults may play a role in the induction and maintenance of the inflammaging process, cognitive performance, and frailty [58]. The composition of gut microbiota is readily modified by diet [59,60,61,62]. Consistent with this observation, high consumption of food rich in anti-inflammatory compounds (e.g., polyphenols) has demonstrated a positive effect on gut microbiota [63]. By contrast, a Western-type diet rich in fat, sugar, and processed foods and low in fiber may lead to a decrease in gut-beneficial bacteria [63]. The NU-AGE trial, with the objective of reducing inflammaging and preventing cognitive decline in apparently healthy subjects, found that the Mediterranean diet approach may prevent cognitive decline [64]. Additionally, an increased intake of fresh fruits, nuts, seeds, and peanuts (important sources of polyphenols and compounds with anti-inflammatory properties) has been associated with cognitive function, probably through modulating gut intestinal microbiota [65] and suppression of neuroinflammatory process by inhibiting free radicals [45]. Dietary lignans are converted through gut bacteria into enteric lignans, a family of polyphenols with therapeutic activity, including anti-inflammatory and apoptotic effects [66]. Urinary enterolignans may be potential markers for microbiota diversity and have been directly associated with dietary inflammatory potential using the DII [67]. However, further studies are warranted to deepen understanding of the association between pro-inflammatory diets and microbiota. 

When we analyzed the population according to the main characteristics predisposing to inflammation, we observed differences by sex and BMI categories. In our study, the association between DIS and biological aging was stronger in women than in men. In previous studies, some diseases, such as inflammatory bowel disease and autoimmune diseases with a strong inflammatory component, were more prevalent in females than in men [68,69]. This differential association between men and women could be explained through differences in sex hormones [70], gastrointestinal characteristics, body composition [71], and differences in gut microbiota [72]. Earlier lifestyle may play a role in aging in adulthood [73] To fully understand the role of the cumulative effect of dietary and lifestyle patterns with biological aging would require access to longitudinal data. 

The DII has been associated with biomarkers of inflammation [50] and with an increased risk of comorbidities hypothesized to be related to inflammation [74]. Diabetes has been associated positively with high DII scores [75,76] and many neurological symptoms that may indicate an acceleration in cerebral aging [77]. The DIS literature, though much smaller, has been associated with inflammation-related diseases, such as sporadic colorectal adenoma [38] and colorectal cancer [78], and with all-cause mortality, including cardiovascular disease and cancer [37]. In the present analysis, “apparently healthy” people (i.e., without evident or self-reported comorbidities) were more likely to have pro-inflammatory diets. Additionally, the subgroup analysis among healthy participants suggested a stronger association between E-DII, DIS, and accelerated biological aging. These results should be considered in light of the cross-sectional design, which may suffer from reverse causality bias. It is conceivable that people with no obvious comorbidities may have a lower perception of risk and, consequently, be more likely to indulge in unhealthy dietary behaviors and other aspects of a lifestyle than people with comorbidities [79].

### Strengths and Limitations

To the best of our knowledge, no analysis has been conducted to link the inflammatory potential of the diet and accelerated biological aging. We uniquely evaluated two different methods to estimate the inflammatory potential of the diet and biological aging through an innovative, deep learning-based measure of BA using circulating biomarkers. Additionally, our results support the use of DIS as an accurate tool for studying associations with the inflammatory potential of the diet, using data from FFQs in observational studies. However, its use would be limited to populations with patterns of intakes similar to those observed in Europe and North America—and not those in other regions such as East, South, and Southeast Asia and East, West, and South Africa. The DII was designed for use in all these populations, including the USA, Bahrain, Denmark, India, Kapan, New Zealand, Taiwan, South Korea, Mexico, and the UK [27]. As of publication, the DII or E-DII has been used in over 900 studies in over 70 countries around the world—vastly more than have used the DIS.

Despite this study’s strengths, several limitations need to be acknowledged. First, in a cross-sectional design, a causal association cannot be established, and reverse causation must be considered. Longitudinal studies are needed in the future to help clarify these aspects. Second, although we adjusted for an extensive list of lifestyle and risk factors, residual and unmeasured confounding cannot be fully excluded. Third, dietary data collected through an FFQ may lead to recall and measurement bias, e.g., lack of accuracy in reported portion sizes and in food composition tables. We partially mitigated this limitation by the exclusion of participants with implausible energy intakes and by energy adjustment [80,81]. Our findings have uncertain generalizability because the cohort originated from a southern Italian region. However, the main characteristics of the Moli-sani cohort are comparable with those in the Italian Cardiovascular Epidemiological Observatory, representative of the Italian population [82].

## 5. Conclusions

Results from a Mediterranean cohort indicate that a pro-inflammatory diet, evaluated through two diverse indices, is directly associated with blood-based markers of biological aging. Putative mechanisms include the low content of polyphenols, antioxidants, and compounds in food that characterize pro-inflammatory diets and their adverse effects on gut microbiota and oxidative damage. Longitudinal analyses are warranted to confirm our results and to test whether biological aging could be on the pathway between pro-inflammatory diets and increased risk of inflammation-related diseases that was previously documented in other cohorts [27,28,29]. 

## Figures and Tables

**Table 1:** Selected characteristics of the participants in Moli-sani sub-cohort (n = 4510) across quarters of the Energy-adjusted Dietary Inflammatory Index (E-DII) and the Dietary Inflammation Score (DIS) ^1^.

		E-DII^TM^ Quartile			DIS Quartile		
Characteristics ^2^	All (n = 4510)	1 (n = 1127)	4 (n = 1128)	*p*-value *	1 (n = 1128)	4 (n = 1127)	*p*-value **
Chronological age, y	55.6 (11.6)	58.4 (11.4)	51.9 (10.9)	<0.0001	57.4 (11.0)	52.0 (11.1)	<0.0001
Biological age, y	54.8 (8.6)	56.3 (8.6)	53.1 (8.3)	0.21	55.2 (8.3)	52.9 (8.3)	0.0012
Δage (BA-CA)	−0.77 (7.7)	−2.1 (7.6)	1.1 (7.4)	0.21	−2.1 (7.4)	0.8 (7.6)	0.0012
Men, %	48.0	37.7	54.0	<0.0001	47.4	47.3	0.003
Education, %				0.11			<0.0001
Lower secondary	52.8	54.1	48.3		49.2	52.7	
Upper secondary	35.0	33.1	38.3		35.0	35.6	
Post-secondary	12.1	12.8	13.4		15.8	11.7	
Missing data	0.02	0.0	0.0		0.0	0.0	
Housing tenure, %				0.19			0.0007
Rent	9.2	8.8	10.8		7.9	12.0	
One dwelling ownership	81.2	80.5	81.9		80.1	80.7	
>1 dwelling ownership	9.5	10.7	7.1		12.0	7.4	
Missing data	0.1	0.0	0.2		0.0	0.0	
Place of residence, %				0.04			0.002
Urban	32.7	70.5	65.7		71.9	65.2	
Rural	67.3	29.5	24.3		28.1	34.8	
Smoking status, %				0.04			0.0002
Non-smoker	49.8	52.1	45.5		47.9	49.1	
Smokers	22.4	17.5	29.0		18.7	28.7	
Former	27.7	30.3	25.5		33.1	22.2	
Missing data	0.1	0.1	0.0		0.3	0.1	
Body mass index, kg/m^2^	28.2 (4.8)	28.6 (4.9)	27.7 (4.7)	0.02	28.4 (4.6)	27.8 (5.0)	0.16
Body weight status, kg/m^2^				0.003			0.02
Normal weight (≤25 kg/m²), %	26.7	24.5	31.6		24.6	32.6	
Overweight (25–30 kg/m²), %	42.0	40.3	42.1		42.9	38.1	
Obesity (≥30 kg/m²), %	31.2	35.0	26.2		32.4	29.3	
Missing data	0.1	0.2	0.1		0.2	0.0	
Leisure-time physical activity, METS hr/d	3.5 (4.0)	3.8 (4.2)	3.1 (3.6)	<0.0001	4.2 (4.4)	2.9 (3.4)	<0.0001
Post-menopausal, %	59.5	70.1	48.2	0.38	68.2	46.7	0.74
Postmenopausal hormone therapy, %	1.4	4.9	1.8	0.02	3.7	1.9	0.03
Comorbidities, %							
Cardiovascular disease	5.5	8.3	4.5	<0.0001	6.6	4.1	0.53
Cancer	3.2	4.4	2.8	0.63	3.7	2.7	0.93
Diabetes	4.9	9.0	2.7	<0.0001	7.3	2.0	0.003
Hypertension	29.6	35.2	22.8	0.54	31.7	21.9	0.11
Hyperlipidemia	7.9	12.1	4.4	<0.0001	11.0	3.8	0.0002
Comorbidity				0.0004			0.01
Without comorbidity	63.1	53.4	71.9		57.8	73.6	
1 or more comorbidities	36.9	46.6	28.1		42.2	26.4	
Dietary characteristics							
Energy intake/d, kcal	2083.7 (576.5)	1843.9 (529.9)	2274.7 (574.7)	<0.0001	2018.2 (580.7)	2233.6 (593.0)	<0.0001
Energy of carbohydrates; %	48.5 (6.9)	48.5 (6.8)	48.7 (7.0)	0.35	47.9 (7.0)	49.8 (7.0)	<0.0001
Energy of fats, %	33.2 (5.6)	34.3 (5.3)	33.2 (5.8)	<0.0001	34.4 (5.6)	32.3 (5.7)	<0.0001
Vegetables; g/d	159.5 (71.6)	198.8 (86.8)	128.9 (51.8)	<0.0001	210.1 (87.9)	122.8 (52.2)	<0.0001
Fruits; g/d	357.7 (204.9)	492.3 (254.8)	227.2 (121.0)	<0.0001	528.2 (255.2)	242.6 (129.5)	<0.0001
Fiber; g/d	20.4 (6.6)	23.3 (7.7)	17.7 (5.3)	<0.0001	24.6 (7.7)	18.5 (5.8)	<0.0001

^1^ Higher E-DII or DIS indicate more proinflammatory diet. ^2^ Presented as means (SD) unless otherwise specified. * *p*-value adjusted for gender and chronological age. ** *p*-value adjusted for gender, chronological age, and energy intake.

**Table 2:** Beta-coefficients ^1^ (95% CI) for biological aging (Δage) according to the E-DII and DIS ^2^, Moli-sani sub-cohort (n = 4510).

	Biological Aging (Δage) ^3^	
	E-DII^TM^	DIS ^4^
Age and sex adjusted	0.14 (−0.03, 0.31)	0.26 (0.09, 0.43)
Multivariable ^5^	0.22 (0.05, 0.38)	0.27 (0.10, 0.44)

^1^ Derived from multivariable linear models. ^2^ Energy-adjusted Dietary Inflammatory Index = E-DII; Dietary Inflammation Score = DIS; Higher E-DII or DIS indicate more proinflammatory diet. For comparison purposes, both scores were standardized. ^3^ ΔAge > 0 suggests accelerated biological aging of an organism compared to its chronological age, while Δage < 0 indicates decelerated biological aging. ^4^ Models for DIS were additionally adjusted by total energy. ^5^ Adjusted for age, sex, education (lower secondary, upper secondary, post-secondary), smoking (non-smoker, smoker, former), BMI, urban, housing (rent, one- or >1 dwelling ownership), leisure-time physical activity (METs h/d), CVD, cancer, diabetes, hypertension, hyperlipidemia, hormonal therapy, and menopausal status.

**Table 3:** Biological aging (Δage) ^1^ by subgroup analyses according to E-DII and DIS ^2^, in the Moli-sani sub-cohort (n = 4510).

		Biological Aging (Δage) ^3^			
		E-DII^TM 4^		DIS ^4,5^	
	n	β (95%CI)	*p*-Value for Interaction	β (95%CI)	*p*-Value for Interaction
Sex					
Men	2164	0.22 (−0.04, 0.48)	0.81	0.08 (−0.17, 0.33)	0.03
Women	2346	0.23 (0.02, 0.45)		0.43 (0.21, 0.65)	
Age groups, y					
≤54.3	2255	0.36 (0.11, 0.61)	0.91	0.48 (0.25, 0.72)	0.39
>54.3	2255	0.46 (0.17, 0.74)		0.33 (0.03, 0.63)	
Body weight status, Kg/m²					
Normal weight (BMI ≤ 25)	1210	0.27 (−0.05, 0.60)	0.01	0.37 (0.05, 0.69)	0.33
Overweight (25 < BMI < 30)	1891	0.25 (−0.01, 0.51)		0.22 (−0.04, 0.49)	
Obesity (BMI ≥ 30)	1409	0.11 (−0.18, 0.41)		0.16 (−0.14, 0.45)	
Smoking status					
Non- smokers	2248	0.15 (−0.08, 0.38)	0.79	0.29 (0.06, 0.53)	0.17
Smokers	1011	0.55 (0.20, 0.91)		0.58 (0.24, 0.93)	
Former	1251	0.09 (−0.24, 0.42)		−0.03 (−0.36, 0.30)	
Comorbidities					
Without	2848	0.29 (0.08, 0.49)	0.11	0.32 (0.12, 0.51)	0.36
1 or more	1662	0.05 (−0.25, 0.34)		0.17 (−0.14, 0.49)	

^1^ β-coefficients (95% CI) derived from multivariable linear models. ^2^ Energy-adjusted Dietary Inflammatory Index = E-DII; Dietary Inflammation Score = DIS; Higher E-DII or DIS indicate more proinflammatory diet. ^3^ ΔAge > 0 suggests accelerated biological aging of an organism compared to its chronological age, while Δage < 0 indicates decelerated biological aging. ^4^ Adjusted for age, sex, education (lower secondary, upper secondary, post-secondary), smoking (non-smoker, smoker, former), BMI, urban, housing (rent, one- or >1 dwelling ownership), leisure-time physical activity (METs h/d), CVD, cancer, diabetes, hypertension, hyperlipidemia, hormonal therapy, and menopausal status, when not stratified for. ^5^ Models for DIS were additionally adjusted by total energy.

## Data Availability

The data underlying this article will be shared on reasonable request to the corresponding author. The data are stored in an institutional repository (https://repository.neuromed.it), and their access is restricted by ethical approvals and the legislation of the European Union.

## Supplementary Materials

The following supporting information can be downloaded at: https://www.mdpi.com/article/10.3390/nu15061503/s1, Table S1: Components, descriptions, and weights of the components of the dietary (DIS) inflammation score in the Moli-sani study; Table S2: Sensitivity analysis for biological aging (Δage) ^1^ excluding comorbidity according to E-DII^TM^ and DIS, in the Moli-sani sub-cohort; Table S3: Sensitivity analysis for biological aging (Δage) 1 for different cut-off point of age according to E-DII and DIS 2, in the Moli-sani sub-cohort.
