# Supplementary material for: Association between the Inflammatory Potential of the Diet and Biological Aging: A Cross-Sectional Analysis of 4510 Adults from the Moli-Sani Study Cohort
Source: Nutrients. 2023 Mar 21;15(6):1503. doi: 10.3390/nu15061503 (PMC10056325; doi:10.3390/nu15061503)
Supplement: Supplementary file 1 [file nutrients-15-01503-s001.zip › nutrients-2254408-supplementary.pdf]

Table S1. Components, descriptions, and weights of the components of the dietary (DIS) inflammation score in the Moli-sani study.

Components	Description	Weights
DIS components[1]		
Leafy greens and cruciferous vegetables	Lettuce, broccoli, Brussels sprouts, cauliflower, turnip green, cabbage, kale, and spinach	-0.14
Tomatoes	Tomatoes, and tomato sauce	-0.78
Other vegetables	Peppers, onions, artichokes, celery, mushrooms, eggplant, and beets	-0.16
Apples and berries	Apple, pear, strawberries, and cherry	-0.65
Deep yellow or orange vegetables and fruit	Peach, cooked and row carrots, squash, and figs	-0.57
Other fruits and real fruit juices	Bananas, kiwi, oranges, grapefruits, mandarins, grapefruit, plums, melon, kaki, fruit juices, and orange juices	-0.16
Legumes	Beans, lentils, and chickpeas	-0.04
Fish	Shellfish, shrimps and crustaceous, dried fish (i.e., salted codfish), canned fish and other fish not included in the previous categories (i.e., salmon, swordfish, anchovies, etc.)	-0.08
Poultry	Chicken or turkey with and without skin	-0.45
Red and organ meats	Hamburger, beef, pork, mutton, lamb, and offal	0.02
Processed meats	Canned meat, and processed meat	0.68
High-fat dairy	Whole and semi-skimmed milk, whole milk, fruit yogurt, ice cream and hard cheese.	-0.14
Low-fat dairy	Low-fat cheese, ricotta cheese, and low-fat yogurt.	-0.12
Added sugars	Chocolate, candies, dried fruits, jams, canned fruits, dry cakes, sweetened carbonated beverages, and non-carbonated fruit drinks	0.56
Coffee and tea	Coffee (decaffeinated and caffeinated), and tea	-0.25
Refined grains and starchy vegetables	Breakfast cereals, bread, rice, pasta, potatoes, and home-baked or ready-made cake	0.72
Nuts	Walnuts, hazelnuts, almonds, and peanuts	-0.44
Fats	Butter, gravy, margarine, and mayonnaise or other creamy dressing	0.31
Supplement score[2]	Ranked score of supplements: vitamins A, B, C, D, multivitamins, B-complex vitamins and iron	-0.80

[1]Dietary components were standardized by sex, to a mean of zero and SD of 1.
[2]All individual supplements were dummy variables, coded as "1" for consumption category and "0" for no-consumption for anti-inflammatory supplements (vitamins A, B, C, D, multivitamins, B-complex vitamins) and "0" or "-1" for pro-inflammatory supplements (iron).
[3]All lifestyle components were dummy variables, coded as 1 for the non-referent category and 0 for the referent category.

Table S2. Sensitivity analysis for biological aging (Δage)[1] excluding comorbidities according to E-DII and DIS[2], in the Moli-sani sub-cohort.

		Biological aging (Δage)[3]	
	n	E-DII	DIS[5]
All the population[4]	4,510	0.22 (0.05, 0.38)	0.27 (0.10, 0.44)
Exclusions			
CVD	4,262	0.29 (0.12, 0.47)	0.29 (0.13, 0.46)
Cancer	4,366	0.29 (0.12, 0.47)	0.26 (0.09, 0.43)
Diabetes	4,289	0.27 (0.10, 0.45)	0.26 (0.09, 0.43)
Hypertension	3,175	0.34 (0.15, 0.54)	0.29 (0.10, 0.48)
Hypercholesterolemia	4,154	0.32 (0.14, 0.50)	0.31 (0.14, 0.48)

[1]βeta-coefficients (95% CI) derived from multivariable linear models.
[2]Energy-adjusted Dietary Inflammatory Index = E-DII; Dietary Inflammation Score = DIS; Higher E-DII or DIS indicate more proinflammatory diet.
[3]ΔAge > 0 suggests accelerated biological aging of an organism compared to its chronological age, while Δage < 0 indicates decelerated biological aging
[4]Adjusted for age (continuous), sex, education (lower secondary, upper secondary, post-secondary), smoking (non-smoker, smoker, former), BMI, urban, housing (rent, one- or >1 dwelling ownership), leisure-time physical activity (METs h/d), CVD, cancer, diabetes, hypertension, hyperlipidemia, hormonal therapy and menopausal status.
[5]Models for DIS were additionally adjusted by total energy.

Table S3. Sensitivity analysis for biological aging (Δage)[1] for different cut-off point of age according to E-DII and DIS[2], in the Moli-sani sub-cohort.

	Biological aging (Δage)[3]				
		E-DII[4]		DIS[4,5]	
	n (%)	β(95%CI)	P-value for interaction	β(95%CI)	P-value for interaction
**Age groups, y**					
<65	3466 (76.8)	0.24 (0.06, 0.42)	0.0057	0.63 (0.41, 0.84)	0.078
≥65	1044 (23.2)	0.11 (-0.28, 0.51)		0.22 (-0.22, 0.66)	
**Age groups, y**					
<70	3900 (86.5)	0.22 (0.04, 0.39)	0.013	0.62 (0.40, 0.83)	0.40
≥70	610 (13.5)	0.02 (-0.48, 0.53)		0.42 (-0.16, 1.01)	

[1]βeta-coefficients (95% CI) derived from multivariable linear models.
[2]Energy-adjusted Dietary Inflammatory Index = E-DII; Dietary Inflammation Score = DIS; Higher E-DII or DIS indicate more proinflammatory diet.
[3]ΔAge > 0 suggests accelerated biological aging of an organism compared to its chronological age, while Δage < 0 indicates decelerated biological aging.
[4]Adjusted for age, sex, education (lower secondary, upper secondary, post-secondary), smoking (non-smoker, smoker, former), BMI, urban, housing (rent, one- or >1 dwelling ownership), leisure-time physical activity (METs h/d), CVD, cancer, diabetes, hypertension, hyperlipidemia, hormonal therapy and menopausal status, when not stratified for.
[5]Models for DIS were additionally adjusted by total energy.
